# *Emesopsis infenestra* Tatarnic, Wall & Cassis, 2011 (Heteroptera: Reduviidae), genus and species new to New Zealand

**DOI:** 10.3897/BDJ.1.e1004

**Published:** 2013-11-06

**Authors:** Stephen E. Thorpe

**Affiliations:** † School of Biological Sciences (Tamaki Campus), University of Auckland, Auckland, New Zealand

**Keywords:** *Emesopsis infenestra*, Reduviidae, New Zealand, Auckland, NZOR

## Abstract

*Emesopsis infenestra* Tatarnic, Wall & Cassis, 2011 (Heteroptera: Reduviidae) is reported from New Zealand for the first time, based on a single specimen collected alive in the wild in Auckland in June 2013. The species was previously known only from Australia (Queensland) and the Loyalty Islands (New Caledonia).

## Introduction

*Emesopsis infenestra* Tatarnic, Wall & Cassis, 2011 was originally described from Australia (2 specimens, including holotype) and the Loyalty Islands (2 specimens). Nothing else has been published about it. As far as I am aware, it has never before been collected from New Zealand.

## Taxon treatments

### Emesopsis infenestra

Tatarnic, Wall & Cassis, 2011

#### Materials

- **Type status:** Other material. **Occurrence:** recordedBy: Stephen E. Thorpe; individualCount: 1; **Location:** country: New Zealand; stateProvince: Auckland; verbatimLocality: Tamaki Campus (East), suburb of Saint Johns; verbatimLatitude: 36.88685S; verbatimLongitude: 174.85260E; **Event:** eventDate: 2013-06-10; **Record Level:** institutionCode: Auckland Museum (AMNZ)

#### Description

**Figure 1.**
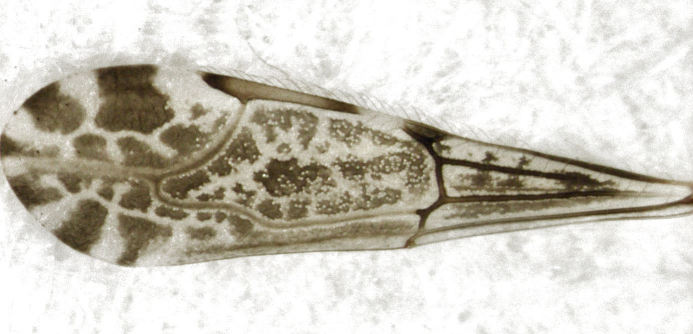
*Emesopsis infenestra* (forewing).

**Figure 2.**
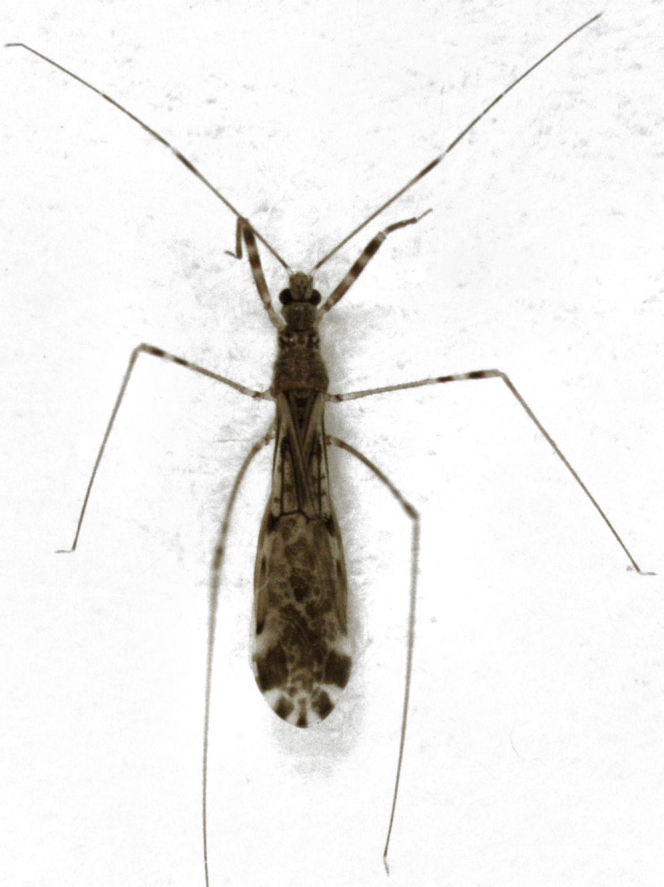
*Emesopsis infenestra* (dorsal habitus, body length about 4.5 mm).

On 10 June 2013, I collected a single specimen of an emesine reduviid amongst long grass in a weedy overgrown wasteland area within the Tamaki Campus (East) of the University of Auckland. It is easily identified as *Emesopsis infenestra* from the original description (Tatarnic et al. 2011), and Nik Tatarnic (pers. comm.) agrees with my determination. Fig. 1 illustrates the distinctive forewing, which is most unlike that of any known species from New Zealand. Fig. 2 illustrates the dorsal habitus of the specimen. Although no further specimens have yet been collected, the chances of it being a post border interception are remote indeed, as are the chances of capture of such a tiny insect if it were an isolated vagrant. Currently, according to NZOR (http://demo.nzor.org.nz/names/4330e783-5a90-4552-8427-e0bf56a027c3), the New Zealand fauna of Reduviidae comprises species of the genera *Empicoris*, *Ploiaria* and *Stenolemus*. I therefore recommend that *Emesopsis infenestra* be added to the New Zealand Organisms Register (NZOR) as present in the wild. Single specimen records can be problematic, but this is largely because most of them are processed long after they were collected, as part of routine curation, and so there is always the possibility of mislabelling with other samples. In this case, however, I personally processed and photographed the specimen within hours of having captured it, so I am confident that there is no possibility whatsoever of mislabelling or contamination. The biostatus (indigenous or exotic) of the species in New Zealand is uncertain. On the one hand, the specimen was found in a highly anthropogenic habitat, which argues for an exotic origin. On the other hand, since the sample size is so low and the species widespread (i.e., 2 specimens from Australia, 2 from the Loyalty Islands and 1 from New Zealand), it is impossible to infer very much at all, and so the species might well be indigenous to New Zealand.

As an aside, there is another unrecorded and as yet unidentified emesine species present in New Zealand. I collected a single specimen in Auckland Domain about 8 years ago and deposited in the New Zealand Arthropod Collection (NZAC). As I no longer have access to NZAC, I cannot check the details, but it was a large species, similar to the native *Ploiaria antipoda*, but fully macropterous, and clearly different to any of the known species in New Zealand. I found it crawling up a spider web covered tree trunk at night.

## Supplementary Material

XML Treatment for Emesopsis infenestra
